# Bisphosphonate-Based Conjugates and Derivatives as Potential Therapeutic Agents in Osteoporosis, Bone Cancer and Metastatic Bone Cancer

**DOI:** 10.3390/ijms22136869

**Published:** 2021-06-26

**Authors:** Zintle Mbese, Blessing A. Aderibigbe

**Affiliations:** Department of Chemistry, Alice Campus, University of Fort Hare, Alice 5700, South Africa; 201208394@ufh.ac.za

**Keywords:** Osteoporosis, bisphosphonates, bone cancer, metastatic cancer

## Abstract

Metastatic bone cancer occurs in every type of cancer but is prevalent in lung, breast, and prostate cancers. These metastases can cause extensive morbidity, including a range of skeletal-related events, often painful and linked with substantial hospital resource usage. The treatment used is a combination of chemotherapy and surgery. However, anticancer drugs are still limited due to severe side effects, drug resistance, poor blood supply, and non-specific drug uptake, necessitating high toxic doses. Bisphosphonates are the main class of drugs utilized to inhibit metastatic bone cancer. It is also used for the treatment of osteoporosis and other bone diseases. However, bisphosphonate also suffers from serious side effects. Thus, there is a serious need to develop bisphosphonate conjugates with promising therapeutic outcomes for treating metastatic bone cancer and osteoporosis. This review article focuses on the biological outcomes of designed bisphosphonate-based conjugates for the treatment of metastatic bone cancer and osteoporosis.

## 1. Introduction

**Figure 1:**
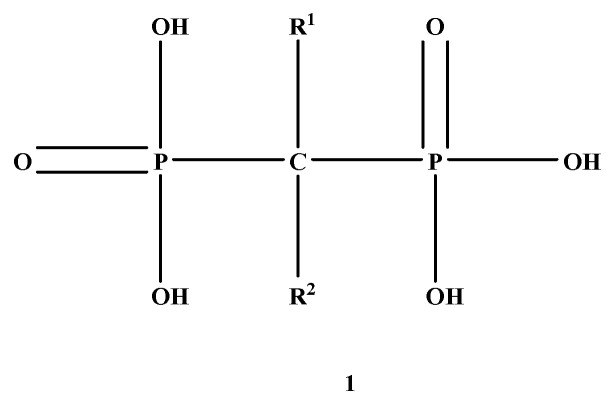
Structure of Bisphosphonate.

Bisphosphonates (BPs) are a significant class of medications that are utilized in the treatment of diseases linked with low bone mass, including Paget’s disease, osteoporosis, and the inhibition of skeletal-related events (SRE) in individuals with osteolytic bone metastases and multiple myeloma [1,2]. BP was synthesized for the first time in the 1800s. Its structure in Figure 1 is composed of two phosphate groups binding to a carbon atom (P-C-P), and their potential to bind to the bone surface is dependent on the P-C-P bond. This bond also improves their binding to the mineralized bone matrix with a subsequent inhibitory effect on bone resorption [3]. The R1 side chain that contains a hydroxyl group (OH) is attached to the central carbon atom and further enhances its binding affinity [1]. BPs anti-resorptive activity is ascribed to the R2 side chain. The existence of a primary amine (NH_2_) that is attached to the central carbon atom raises strongly anti-resorptive effectiveness [1]. Hence, the addition of hydroxyl group and/or primary amine group increases the affinity for calcium ions, which results in favored localization of the remedies to the sites of bone remodeling [4]. BPs are classified into two groups which are nitrogen-containing bisphosphonates (N-BPs) e.g., pamidronate, neridronate, alendronate, etc., and non-nitrogen containing bisphosphonates (N-NBPs) e.g., etidronate and clodronate [5]. They are also classified into three generations based on their therapeutic effects, as shown in Table 1. The 3rd generation BPs are more effective than the 1st and 2nd generation BPs [5,6]. The N-NBPs are incorporated into the energy pathways of the osteoclast, resulting in disrupted cellular energy metabolism leading to apoptosis. N-BPs exert their effect on osteoclasts via their inhibition of the mevalonate pathways, resulting in the disruption of the intracellular signaling and induction of apoptosis [7].

In the treatment of osteoporosis, BPs are used, and they are active for some pathologies characterized by abnormally high bone resorption, which includes metastatic bone cancer, etc. [8,9]. BPs have experienced a huge development in the treatment of some bone diseases [10,11].

Currently, some of the uses of BPs are:

✓ In corticosteroid and postmenopausal induced osteoporosis, the most utilized BPs in these cases, which block the presence of pathological fractures, is alendronate [12].
✓ They are also utilized to enhance bone morphology and decrease pain in Paget’s disease [13].
✓ In hypercalcaemia of malignancy, its role is in trying to check hypercalcaemia, reducing pain, and preventing the development of osteolitic lesions and fractures [14].
✓ In people with bone metastasis, it is utilized to decrease fractures, hypercalcemia, and relieve pain [15,16].
✓ They are used to decrease associated bone pathologies in multiple myeloma, including pain, fractures, and vertebral collapse [17,18].

Despite the efficacy of BPs in the treatment of many bone-related diseases and their relatively good record, BPs also suffer from several limitations such as diarrhea, esophageal erosion, gastric ulcers, rare cases of esophageal cancer, hepatotoxicity, conjunctivitis and hypocalcemia, bone pain, fever, osteonecrosis of the jaw, and femur fracture [19,20,21,22]. This review focuses on bisphosphonate-based conjugate compounds developed for treating osteoporosis, bone cancer, and cancers that spread to the bones, such as prostate, lung, and breast cancer.

## 2. Osteoporosis

Osteoporosis is a leading public health challenge, and it has become one of the most dominant chronic diseases in the world [23]. It is the most widely recognized skeletal disease that is initiated by an imbalance in bone metabolism, leading to a gradual loss of bone mass and attenuation of bone strength, thus disposing to low-energy fractures [24]. It is common in older adults, more especially postmenopausal women. This disease is characterized by bone loss that results in bone fractures in the spine and hip [25]. In developed countries such as the United States, the number of individuals with osteoporosis is increasing due to the increasingly aged population. Therefore, the treatment and prevention of osteoporosis are of great concern [25]. The estimates of women in danger of osteoporotic fractures and the respective economic burden to the health care systems in the next decades are expected to grow continuously [24]. In 2005, the cost for the treatment of fractures due to osteoporosis in the United States was $16.9 billion; it is predicted that the cost will increase to $25.3 billion by the year 2025 [23]. The pharmacological agents utilized in the management of osteoporosis include medications that precisely inhibit bone loss, decrease the risk of future fractures, and also increases bone strength [26]. In patients with osteoporosis, BPs are known drugs that inhibits the loss of bone mass. The therapeutic efficacy of BPs is based on their remarkable selectivity to the bone rather than other tissues [27]. Many BPs have been approved and utilized to manage osteoporosis, e.g., alendronate, risedronate, zoledronate, ibandronate, etc., in several countries [26,28]. BPs decrease fracture risk by 30–70% at the spine and increase bone mass. Furthermore, they do the same at varying degrees at other skeletal sites in postmenopausal women [28,29].

Risk factors of osteoporosis include:

✓ Age: the people at high risk are individuals who are at the age of 65 and above.
✓ Gender: men are at less risk than women because of the absence of menopause [30].
✓ Family ancestry plays a significant role.
✓ Drinking too much alcohol and smoking are risk factors for osteoporosis [31].

### Bisphosphonates Conjugates for the Treatment of Osteoporosis

**Figure 2:**
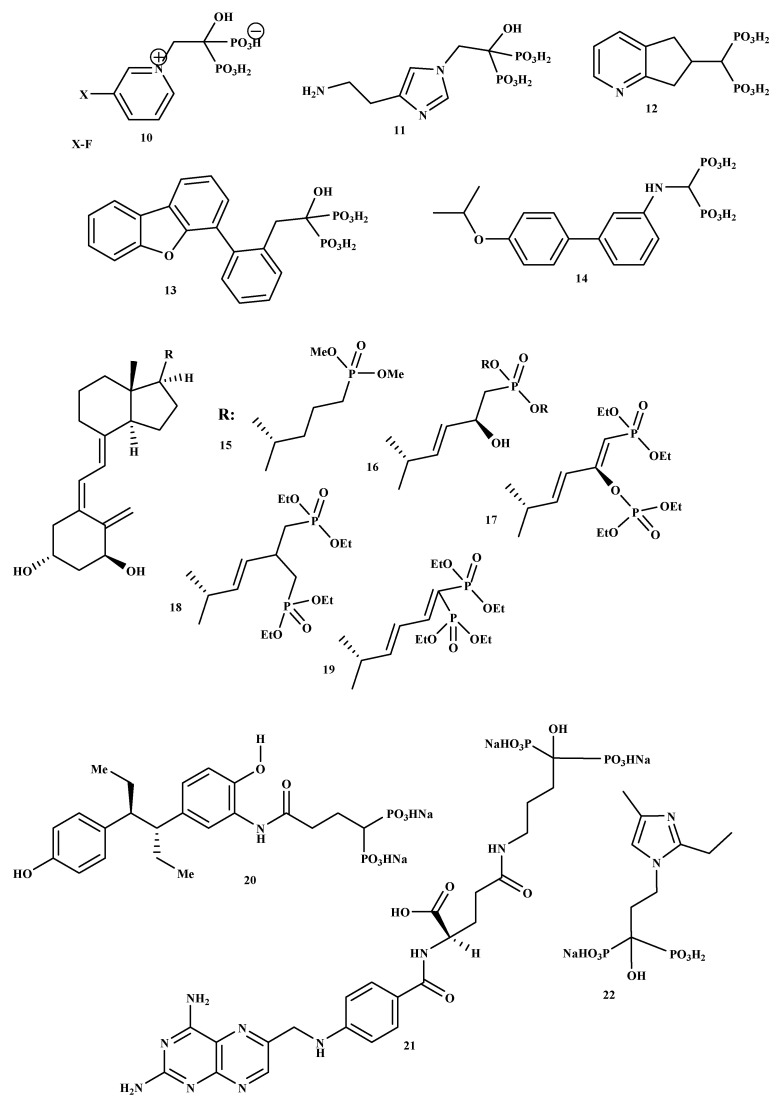
Structures of BP-conjugates **10–22**, potential compounds reported for the treatment of osteoporosis.

New BP-conjugate anti-osteoporotic agents have been designed and are presented in Figure 2. Compounds 10, 11, 12, 13, 14 were not effective compared to the drugs that are already utilized to treat osteoporosis [32]. Therefore, there is still a need to develop drugs that will be effective as anti-resorptive agents for treating osteoporosis. It is reported that vitamin D conjugates for effective for improving good bone health; hence, Steinmeyer et al. synthesized a series of novel vitamin D conjugates by combining bisphosphonate moieties and vitamin D [33]. The in vitro evaluation of vitamin D conjugates (15, 16 and 18, 19) exhibited moderate activities, while 17 showed a highly moderate vitamin D-like activity in HL 60 and ROS 17/2.8 [33]. Tsushima et al. synthesized and evaluated the effects of a new hybrid compound of estrogen-bisphosphonate, 20 [34]. The results showed that 20 is an effective bone-targeting therapeutic, and it also displayed a strong ability to inhibit bone loss. Therefore, utilizing bisphosphonates for hybrid compounds is a promising approach for successful delivery of estrogen to the bone [34]. Xie et al. prepared and characterized a new bone-targeting conjugate, methotrexate-alendronate, 21. ^1^H-NMR, ^13^C-NMR, ^31^P-NMR, and LC-MS were used to characterize the conjugate [35]. The conjugate compound prevented bone resorption and the formation of osteoclast compared to methotrexate and alendronate utilized alone. Compound 21 potential to adhere to the exposed bone surface and improve drug accumulation in the pathological region for targeted therapy against osteoclastogenesis was significant. Therefore, the results revealed methotrexate-alendronate conjugate as a potential therapeutic for the treatment of rheumatoid arthritis and osteoporosis by targeting osteoclastogenesis [35]. Motaleb et al. synthesized and characterized a novel zoledronate conjugate, 22, and successfully radiolabeled with 99mTc [36]. Compound 22 displayed excellent biological activities with high preferential uptake to the bone with clearance from blood, soft tissues; and these results suggest that zoledronate conjugates is a promising drug as an anti-osteoporotic agent [36].

## 3. Bone Cancer

**Figure 3:**
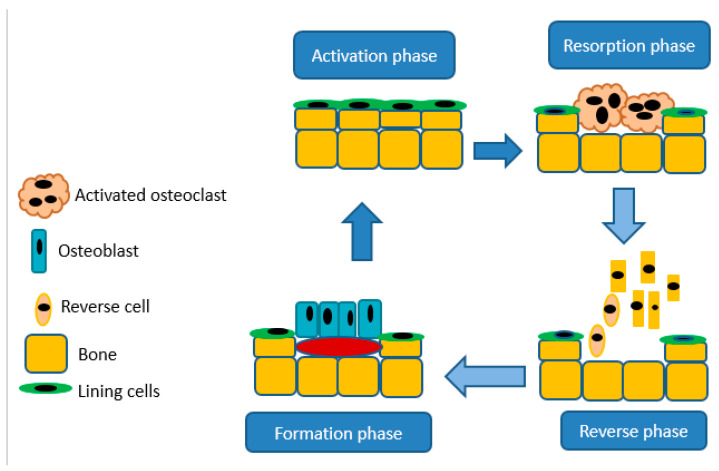
A schematic diagram illustrating bone resorption and bone formation.

Bones are crucial in the body for movement and structure, mineral storage, protection of organs, and in blood cell production, provides a site as bone marrow houses hematopoietic cells for the production of leukocytes, platelets, and red blood cells [37]. Bones are essential for storing phosphorus and calcium in the body because of their hydroxyapatite crystal structure. Bones continuously undergo cycling of resorption and deposition of the osteoid and hydroxyapatite matrix. Bone releases minerals via resorption by the osteoclasts, the cells accountable for separating bone matrix, whereas osteoblasts develop novel bone through emitting chondroitin, osteocalcin, and collagen to formulate osteoid [37]. The balance between bone formation and resorption relies upon signals from chemokines, cytokines, and mechanostimulation. Moreover, resorption by osteoclasts is important for the maintenance of typical bone density. An imbalance in these procedures results in hyperplastic bone, including mutual degenerative bone infections [37]. A schematic illustration of bone resorption and formation is shown in Figure 3.

For cancer patients, it is reported that cancer usually spreads to the bone. Metastatic bone cancer happens mostly in patients with prostate, lung, and breast cancer [38]. The resulting skeletal events include spinal cord compression, pathologic fractures, and bone pain. Metastasis occurs when cancer cells spread from the primary cancer site to an inaccessible site through the circulatory system forming secondary cancer [39]. The reported median survival rate for people with metastatic bone resulting from breast, renal, and prostate cancer ranges from 12 to 33 months [35]. The survival rates for people with primary lung cancer are very low and range from 9.5% to 12% in 12 months [38]. People with bone metastases can experience some limitations in their daily lives, such as increased medical expenses, decreased quality of life, and the threat of survival [40].

Osteosarcoma is a well-known diagnosed primary bone malignancy [41]. Osteosarcoma generally occurs either during active growth phases in adolescence and young adults or often associated with a pre-existing condition, like Paget’s disease (in adults above 65) [41]. The occurrence of bone cancer in males is higher than in females. However, osteosarcoma in females develops earlier compared to males [42]. Metastatic cancer affects the skeleton. In particular, cancers with a high incidence and relatively long clinical course include prostate, lung, and breast cancers, and they often metastasize to the bone [43,44,45]. Most primary cancers and bone metastases exhibit improved osteoclast action and bone resorption [46,47] that can lead to hypercalcemia, pathological fractures, and pain [45]. Therefore, techniques to decrease the incidence and morbidity of bone metastases are obviously of tremendous clinical significance [41]. The treatment that is currently used is the combination of chemotherapeutics and surgery. Thecombination of high dosages of methotrexate, cisplatin, ifosfamide, and doxorubicin have prompted an increased survival rate. Nonetheless, the utilization of anticancer medications is limited by severe side effects. The poor bone blood supply, drug-resistance, and non-specific uptake require the utilization of high toxic doses of anticancer drugs [41]. Hence, there is a need to develop bone-targeted anticancer agents with reduced and/or no side effects for the treatment and prevention of malignant growth-related bone diseases [48].

Numerous therapeutic techniques have been developed for the management of malignant-related skeletal complications. These approaches incorporate either anti-neoplastic intervention, for example, chemotherapy, hormonal therapies, radiotherapy, and topical surgeries, or bone helpful treatment such as calcium, analgesics, and vitamin D supplements [39]. In the treatment of malignant bone metastasis, synthetic anti-resorptive agents are considered to be indispensable [39]. BPs are known as the most effective of these drugs. They have an established role as palliative treatment in people with skeletal metastases. BPs increases the prevention of cancer cell invasion and adhesion to the bone matrix and induces apoptosis of osteoclasts [39]. Some studies reported that BPs stimulate osteoblastic bone formation in vitro and in vivo. BPs is one class of pharmacological agents used for the prevention and management of myeloma bone disease. However, agents like anti-RANKL antibody, Denosumab are also utilized in people with bone metastasis resulting from prostate and breast cancer. Denosumab is currently under clinical trials in patients with Multiple myeloma [49]. Anticancer agents, for example, doxorubicin, cisplatin, and methotrexate were, covalently incorporated into the terminal amino group of the amino-BP alendronate utilizing a suitably bio-reversible amide bond. The BP-conjugates displayed prolonged blood circulation, binds to the hydroxyapatite matrix of the bone, and hence released the BP together with the cytostatic agent into the bone micro-environment via the cleavage of the amide linker of the BP-conjugate [50].

### BP Conjugates as Bone-Targeting Compounds

**Figure 4:**
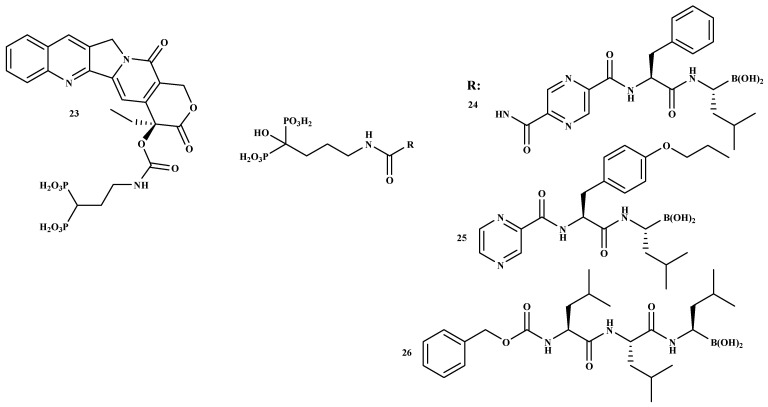
The structures of BP-conjugates **23–26**, compounds with good bone-targeting capability.

The administration of BPs utilized in clinical practice is usually via intravenous or oral routes. Oral administration of BPs is challenging due to poor gastrointestinal tolerability and bioavailability [32]. Due to their high affinity to the bone, between 30% and 60% of the absorbed drug quickly binds to bone mineral. This feature is utilized to build drug BP-conjugates that can be considered a good approach for selective drug targeting to the bone. Such an effective transport of therapeutic agents to the bone is called osteotropic drug delivery system (ODDS). It decreases drug toxicity and enhances its bioavailability at the desired site. The effective transportation of bioactive agents to the bone is named osteotropic drug delivery system [32]. The development of bone metastases is one key reason for tumor-related death. Hence, it is important to design anti-tumor drugs that target bone tissue for enhanced therapeutic outcomes. Polymer-based carriers incorporated with BPs together with anti-tumor agents have been developed for synergistic anticancer effects. Bisphosphonate was conjugated to camptothecin for bone targeting [51]. Additionally, the combination of BPs with anti-cancer drugs, which include bortezomib, doxorubicin, gemcitabine, or camptothecin, has been effective against multiple myeloma cells. Examples of BP-conjugates containing anti-cancer drugs are shown in Figure 4 [51,52]. Agyin et al. prepared a sequence of proteasome inhibitor BP-conjugates that are effective inhibitors of proliferation and cell viability in vitro in myeloma cell lines. Table 2 shows the in vitro anti-proliferative and cytotoxic effect of the bone-targeted proteasome inhibitors of compounds 24, 25, and 26. The IC_50_ values of compounds 24 and 25 were moderate, while the IC_50_ value of compound 26 showed synergistic effects on 5TGM1 cell lines [52]. According to the results, the compounds are potential bone-targeted agents for the treatment of multiple myeloma.

**Figure 5:**
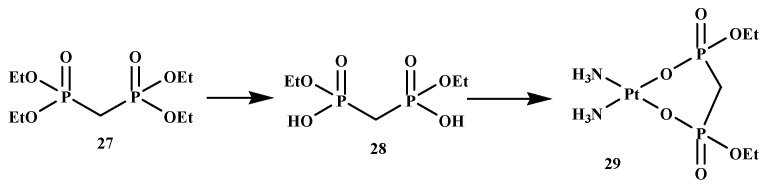
BP-conjugate **29**.

To develop an active agent for the treatment of bone metastases, some BP-conjugates with platinum(II) complexes, such as cisplatin (since it has a broad spectrum range of cancer drugs), have been synthesized and evaluated for the treatment of bone metastases [53]. Nakatake et al. synthesized cis-diammine (P,P-diethyl methylenebisphosphonato) platinum(II) (DEBP-Pt) and intensively evaluated the impact on the cancer cells and bone resorption activity of osteoclast Figure 5. Cell development inhibitory impact of DEBP-Pt (21) was examined against four cancer cell lines. The results in Table 3 revealed that compound 29 displayed anticancer activity, and the impact of compound 29 on bone resorption activity was also evaluated. Compound 29 demonstrated a prominent inhibitory impact on bone resorption, as displayed in Table 4. The results indicated the anticancer activity and bone resorption inhibitory effect of the compound. Therefore, the outcome of the results reveals the potential of compound 29 for the treatment of metastatic bone cancer [53].

**Figure 6:**
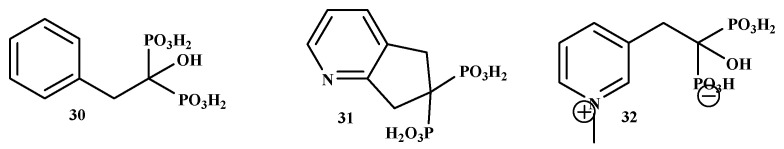
The structures of BP-conjugates **30–32,** risedronate containing compounds.

Ebetino et al. synthesized BP-conjugates as shown in Figure 6. The phenyl conjugate NE-58022 (30), NE-58086 (31) of risedronate has a coupling mode almost the same as that of risedronate. However, it was less effective with IC_50_ values of 1626, 2588 nM (IC_50_ 5.7 nM for risedronate), respectively. NE-10575 (32) was an effective conjugate with an IC_50_ value of 19.6 nM. According to the results, compound 32 is a potential bone antitumor drug [54].

**Figure 7:**
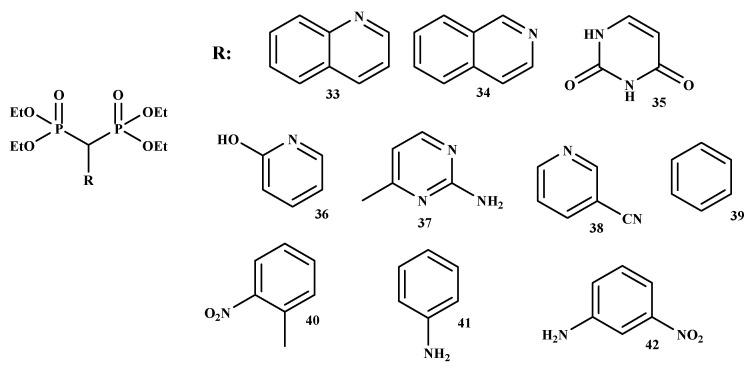
The structures of BP-conjugates **33–42** reported by Kunda et al. [55].

Kunda et al. synthesized new BP-conjugates (33–42) (Figure 7) using NaH/PEG with a good percentage yield ranged between 59% and 71% [55]. All the conjugates were purified by column chromatography and characterized by IR, ^1^H-, ^31^P-NMR, and MS spectroscopy.

**Figure 8:**
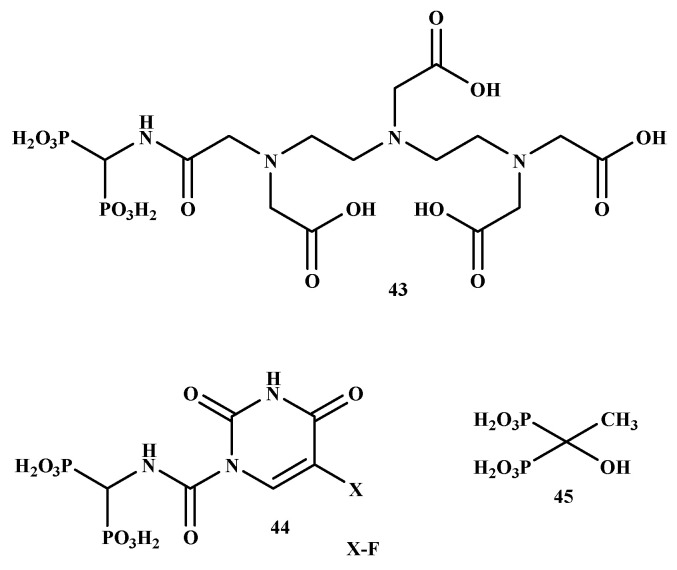
The structures of BP-conjugates **43–45** reported by El-Mabhouh et al. [56].

In another study, it was proposed that the DTPA/BP chelate would allow medicinal radionuclides to target the bone. In contrast, the 5-fluorouracil BP-conjugate might be specifically transported to metastases bone without systemic toxicity. El-Mabhouh et al. prepared ^188^Re-5-FU/BP (43) and ^188^Re-DTPA/BP (44) with good stability and high radiochemical yields, as shown in Figure 8 [56]. The compounds’ high bone uptake, particularly in the joints, low soft-tissue uptake, and rapid plasma clearance, was significant. This increases the potential of specific treatment to metastatic bone lesions while avoiding complete toxicity. Therefore, BP-conjugates are promising therapeutics for the management of bone metastases [56].

**Figure 9:**
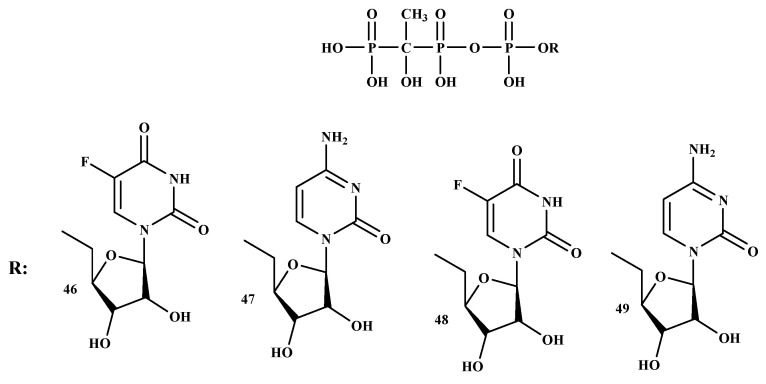
BP-conjugates **46–49,** potential compounds for the treatment of Tumor-induced bone disease.

Tumor-induced bone disease (TIBD) is one of the significant reasons for mortality and morbidity in people with malignant growth. The development of an active drug for the treatment of TIBD is necessary because it causes extensive morbidity [52]. TIBD is common in cancer patients and a median survival of less than two years. The development of BP-conjugates with improved anti-resorptive and cytotoxic is a pressing need to enhance the medication for patients with TIBD. BP-conjugates can be utilized effectively as bone-targeting therapeutics. In addition, BP-conjugates can enhance the control of disease-associated symptoms, reduce the intrusion of remedy on the events of everyday life, and reduce treatment-related toxicity [57]. BP-conjugates, (MBC-1 (46), MBC-11 (47), MBC-9 (48), and MBC-29 (49)) as shown in Figure 9. The results showed that compound 47 can be delivered to the bone and it displayed anti-tumor activity with prolonged survival rate, making it a promising therapy for TIBD [57].

## 4. Metastatic Cancer That Spreads to the Bones

Bone metastasis is linked with most types of cancers leading to high mortality and morbidity in cancer patients. However, it is most prevalent in people living with lung, breast, and prostate cancer [58,59]. Although bone metastasis is common in myeloma, prostate, and breast cancer, according to radiographic appearance, the primary pathogenesis often includes both osteolytic and osteoblastic processes [60]. About 75% of patients suffer from severe pain during the time of diagnosis of metastatic bone. It may lead to SREs, such as hypercalcemia, pathological fractures, and spinal cord compression resulting in increased medical care cost, shortened survival rate, and decrease quality of life [59]. Some researchers reported that BPs could prevent tumor cell invasion, angiogenesis, proliferation and enhance survival rate in vivo [61]. BPs have also been utilized to minimize pain in multiple myeloma and reduce skeletal complications [62]. In addition, BPs can slow down bone-resorption and exert cytotoxic effects on osteoclasts [63]. In people with cancer, BPs can affect the bone micro-environment to decrease the invasion of the cancer cells [64]. BPs also exhibit anti-angiogenic and anti-tumor activity [65,66,67,68,69,70]. However, the biological diversity of bone metastasis and tumors can result in different responses to BP remedy. Moreover, BP drug can expose patients to serious side effects such as osteonecrosis of the jaw, hypocalcemia, and nephrotoxicity [71].

Osteonecrosis of the jaw is one of the most severe side effects. Most cases of bisphosphonate-related osteonecrosis of the jaw have been reported in people with breast cancer and multiple myeloma treated with high doses of intravenous bisphosphonates. Moreover, it has also been reported in people taking bisphosphonates for the treatment of osteoporosis. The risk factors for bisphosphonate-related osteonecrosis of the jaw comprise the usage of higher dose, intravenous bisphosphonates and prolonged duration of administration. Furthermore, the usage of anticancer therapy and glucocorticoids, smoking, cancer, and history of diabetes are additional risk factors [37,72]. Hypocalcemia is also a common problem associated with bisphosphonate usage; moreover, the incidence can be as high as 18%. Hypocalcemia is more common, secondary to intravenous bisphosphonates, and in people with hypoparathyroidism, an underlying untreated vitamin-D deficiency, poor calcium intake, and hypocalcemia. Vitamin D and calcium deficiency should be treated before administering bisphosphonates, especially intravenous bisphosphonates [73,74]. Some BPs such as pamidronate, zoledronate, clodronate, and ibandronate have been in clinical use for the management of health problems associated with tumors that cause osteolysis. However, there is an increasing evidence from preclinical studies that demonstrate BPs good antitumor activity [75,76]. Numerous investigations have revealed a decrease in the risk of colorectal and breast cancer among BP users. Therefore, BPs can be clinically effective for the prevention and treatment of tumors [77].

### 4.1. Breast Cancer

Globally, breast cancer is the leading cause of death amongst women. The World Health Organisation (WHO) reported that over 1.38 million women suffered from breast cancer in 2008 [78]. In 2016, roughly 61,000 cases of non-invasive breast cancer and 246,660 cases of invasive breast cancer were reported by the American Cancer Society (ACS), with 40,730 deaths [78]. It is estimated that between 65% and 75% of patients with breast cancer may suffer from cancer spreading to the bones with problems, such as hypercalcemia, spinal cord compression, and pathological fractures [79]. Strategies to preserve bone health are therefore an important aspect of breast cancer treatment [80]. Treatment approaches for the management of breast cancer are limited. Chemotherapeutic drugs are inhibitors of many metabolic pathways, but there are severe side effects such as fatigue, nausea, loss of appetite, hair loss, etc. [78]. Several studies proved N-BPs to be active therapies for the treatment of bone metastases in breast cancer patients. The oral use of BPs is associated with a reduced risk of colorectal and breast cancers [81]. Furthermore, BPs inhibit SRE and cancer therapy-induced bone loss in bone metastatic breast cancer [82]. Studies in preclinical trials reported the anticancer effect of BPs.

Some epidemiologic research observed a beneficial effect of using BP in inhibiting breast cancer. Hence, numerous clinical trials studied the adjuvant usage of BP in early breast cancer [82]. For the prevention of SREs in postmenopausal women, zoledronate has been confirmed as a suitable bioactive agent. The largest group of patients with breast cancer are postmenopausal women with early-stage breast cancer, under estrogen-depleting treatment [83]. The study of breast cancer in Northern Israel revealed that the utilization of BPs for more than one year was linked with a 28% reduction in the risk of postmenopausal breast cancer, and these observations were confirmed by the Women’s Health Initiative (WHI) for oral BPs. Therefore, BPs reduce the risk of bone metastasis in high-risk breast cancer patients [83]. Pamidronate application in breast cancer has been demonstrated to decrease morbidity by decreasing the progression of bone metastases, reducing the need for radiation and pathological fractures. Similarly, clodronate has been reported to decrease the growth of new bone metastases in breast cancer patients, as well as the number of skeletal events [84].

#### 4.1.1. In Vitro Studies of BPs in Breast Cancer

Alendronate is one of the BPs utilized to reduce the risk of breast cancer in post-menopausal women. Ilyas et al. studied the cytotoxic effect of alendronate on HTB-132 malignant growth cell line. Alendronate was tested at 5, 10, 15, and 20 M, and the results showed a 47% cell death at 5 M, indicating that the treatment and dosage were most active at low concentration [78]. Additionally, alendronate can induce cytotoxic effects in HTB-132 cell lines at low concentrations and can be utilized as chemotherapeutic agents in breast cancer [78]. Body et al. determined the effects of N-BPs on the proliferation of malignant growth cell lines. The IC_50_ value of four N-BPs, which include alendronate, ibandronate, risedronate, and zoledronate, were observed in numerous cancer cell lines. Human cancer cell lines such as the GBM cell line U87, MDA-MD-43, and GBM patient-derived primary cell line SK429 were used. The results showed that zoledronate was effective with the lowest IC_50_ values ranging from 3 μM to 37 μM against all the cancer cell lines. Zoledronate exhibits an effective inhibitory effects on these different human cell lines [85].

#### 4.1.2. Bisphosphonates Conjugates for the Treatment of Breast Cancer

**Figure 10:**
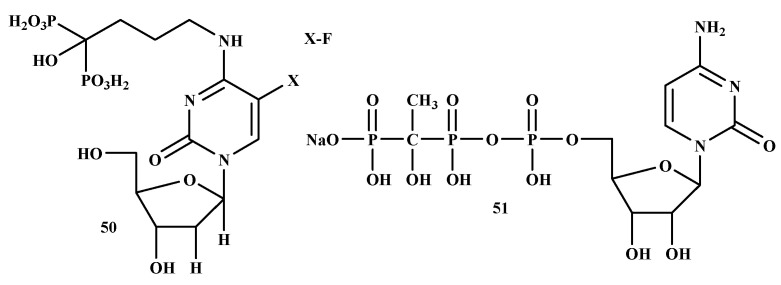
Structures of BP-conjugates with anti-breast cancer activity **50, 51**.

Schott et al. studied the therapeutic efficacy of 5-Fluoro-2′-deoxyuridine-alendronate (50) in breast cancer (Figure 10). Novel antimetabolite-bisphosphonates conjugates (5-Fluoro-2′-deoxyuridine-alendronate) were prepared for bone targeting [50]. The IC_50_ and IC_90_ values of 5-Fluoro-2′-deoxyuridine, 5-fluorouracil, zoledronate, alendronate, and 5-Fluoro-2′-deoxyuridine-alendronate on the cell line tested are shown in Table 5. Compound 42 showed inhibitory effects in MCF-7 cell line with an IC_50_ value of 44.0 μM compared to alendronate and 5-Fluoro-2′-deoxyuridine with an IC_50_ value of 55.9 μM and 51.3 μM, respectively. Compound 50 did not exhibit any inhibitory effects in the tested cell lines compared with 5-Fluoro-2′-deoxyuridine, zoledronate, and alendronate [86]. In conclusion, the study indicates that compound 50 is toxic to osteoclasts in vitro but has an osteoblast and tumor cell-sparing effect compared to alendronate [87].

### 4.2. Prostate Cancer

Bone metastases are the primary reason for morbidity in people with cancers like prostate, lung, breast, and multiple myeloma cancer. Specifically, breast and prostate cancer target the skeleton as the preferred site of dissemination, representing 80% of all bone metastasis [88]. The second most dominant cancer globally is prostate cancer, with more than half a million men worldwide being diagnosed with prostate cancer [89,90]. US Cancer Statistics stated that in 2014, 172,258 new cases were diagnosed, and 28,343 deaths were ascribed to prostate cancer disease in the country. Regardless of the attempt to treat the disease, about 6% of people with prostate cancer will grow skeletal metastasis during disease progression [90,91]. The clinical complications include fractures, pain, hypercalcemia of malignancy, and compression of the spinal cord. The possibility of extensive survival radically reduces when metastases occur since present treatments are relatively ineffectual [88,92,93]. The purpose of treating bone metastasis is to reduce and/or inhibit the complications of SREs, thus improving patients’ quality of life [94]. Though bone metastases in prostate cancer patients are common of the bone-forming osteoblastic type, histological and biochemical analyses proposed that there is also an addition of osteoclast action in these lesions, prompting an increased risk of SREs and bone destruction [87]. Osteoclast inhibition restrains bone metastases in preclinical trials of prostate cancer. Consequently, osteoclast initiation plays a significant role in metastases development [95,96].

Bone metastasis causes negative results in patients with prostate cancer, and 80% of those with advanced prostate cancer have bone metastasis. Radiotherapy is the ideal therapy for bone metastasis. The complications of radiotherapy include pneumonia, myelo-suppression, and pancytopenia [97]. BPs are useful to inhibit fractures, skeletal complications, and bone pain [96]. Some studies showed that osteolysis might be present in bone metastases prostate cancer [98]. BP treatment inhibits possible pathological fractures in men with prostate cancer metastases and reduces pain [97]. Zoledronate is the most potent BP, and it prevents negative results related to the bone in patients with prostate cancer and is linked to secondary bone metastasis [97]. Zoledronate and pamidronate are BPs that are accepted by the US FDA for the treatment and the prevention of the complications of SREs in prostate cancer metastatic patients [94].

#### 4.2.1. In Vitro Studies of BPs in Prostate Cancer

Zoledronate displays effective action of anti-bone resorption and anti-cancer activity [99]. Some in vitro preclinical studies demonstrated that zoledronate prevent tumor cell adhesion, thus damaging the progression of tumor cell metastasis and invasion. Furthermore, it was reported that zoledronate has a significant effect in vitro stimulation on the angiogenesis of *γ*/*δ* T lymphocytes [100,101,102]. The tumor cell apoptosis is one of the crucial anticancer mechanisms of zoledronate [103]. In vitro and in vivo studies revealed a synergistic anti-tumor action of zoledronate when utilized in combination with either targeted molecular agents or cytotoxic drugs [103]. Pamidronate or zoledronate together with farnesyl transferase inhibitor (FTI) R115777 were utilized to assess the effects of the combination therapy on apoptosis and inhibition growth [104]. A synergistic effect was found between zoledronate and R115777 on both androgen-dependent LNCaP and androgen-independent PC3 prostate cancer cell lines, and special effects were due to improve inactivation and apoptosis of Erk and Akt [104]. Moreover, De Rosa et al. reviewed the efficacy of a combination therapy of docetaxel and zoledronate on hormone sensitive prostate cancer cell line, LNCaP. A combination of DTX and ZOL in hormone and drug refractory, PC-3 and DU-145 prostate cancer cells resulted in a significant synergistic effect resulting from inhibited cell growth via the apoptotic pathways by the downregulation of the anti-apoptotic protein Bcl-2 [105].

#### 4.2.2. Bisphosphonates Conjugates for the Treatment of Prostate Cancer

**Figure 11:**
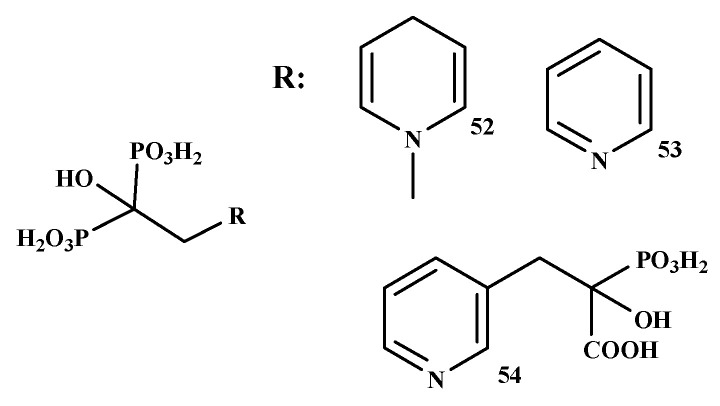
Structures of BP-conjugates with anti-prostate cancer activity **52–54**.

Boissier et al. prepared the BP-conjugate of prostate cancer, as shown in Figure 11. The in vitro inhibitory effects of four BPs (ibandronate, zoledronate, risedronate, and clodronate) and three BP-conjugates (NE-10244 (52), NE-58051 (53) and NE-10790 (54)) were evaluated. Zoledronate, ibandronate, and compound 52 were more effective than compound 53 and compound 54. Compound 52 inhibited tumor cell invasion with an IC_50_ value of 5 × 10^−10^ M. Compound 54 inhibited tumor cell invasion similar to that observed with compound 52, whereas compound 53 did not show any inhibitory effect. Compound 52 inhibited the growth of the PC-3 prostate cancer cell line in vitro [106].

### 4.3. Lung Cancer

Lung cancer is known as one of the leading causes of death worldwide [107,108]. In the United States in 2018, it was reported that lung cancer is accountable for 234,030 new cases and 154,050 deaths. Tobacco smoking is one of the most widely recognized risk factor contributing to lung cancer [109]. Lung cancer can be classified into two types, (1) non-small cell lung cancer (NSCLC), which tends to respond poorly to chemotherapy and radiation therapy; and (2) small cell lung cancer (SCLC)—this group is not common and is categorized by high proliferation rate and also sensitivity to combined chemotherapy and radiation therapy in limited-stage carcinoma cases [109]. Bone metastases development is 30–40% in patients with NSCLC. The high mortality rate is predominantly a result of the difficulties in the early diagnosis of bone metastases and the high-metastatic capability of lung cancer [107]. A study that was conducted in Japan demonstrated that bone metastasis was present at the time of diagnosis in 48% of patients with stage four NSCLC and 40% of those with extensive-stage SCLC [110]. The development of bone metastases in lung cancer can lead to SREs, for example, pathologic fractures, spinal cord compression, hypercalcemia, and radiation therapy [111]. The therapeutic approaches to treat bone metastases are still limited [112]. Hence, it is vital to develop treatment of bone metastases to inhibit SREs [113,114].

In the preclinical studies, BPs anticancer effect against NSCLC, such as invasion, angiogenesis, inhibition of tumor cell proliferation, and micro-metastasis have been reported. Moreover, preclinical studies also revealed that BPs improved the inhibitory effects of EGFR-TKIs on NSCLC with EGFR mutation both in vitro and in vivo [112]. However, BPs associated with nephrotoxicity, need observing and might require initial dose change and withholding of doses [108].

#### 4.3.1. In Vitro Studies of BPs in Lung Cancer

BPs are powerful inhibitors of osteoclastic bone resorption and have been commonly utilized in the management of hypercalcemia. Yano et al. reported a model of various organ metastases with SCLC cell line, which includes SBC-5, in NK3 cell-depleted SCID mice [114]. In the SBC-5 model, the cell metastasizes in multiple organs, which include the liver, lung, kidney, and bone, resembling characteristics of SCLC in humans. A BP, minodronate (YM529), prevents osteolytic bone metastasis through the prevention of bone resorption [114]. Moreover, YM529 anticancer effects on different kinds of cancer cells in vitro and in vivo have been reported [112]. However, YM529 did not extend the survival of cancer-bearing mice because of visceral metastasis [114]. Furthermore, research that demonstrated the impacts of YM529 on NSCLC has been limited. Therefore, the study of YM529 displayed its anticancer activity on NSCLC cell lines in vitro and prompted apoptosis and the G1 arrest cell cycle via down-regulation of phosphorylation of ERK1/2 [115].

#### 4.3.2. Bisphosphonates Conjugates for the Treatment of Lung Cancer

**Figure 12:**
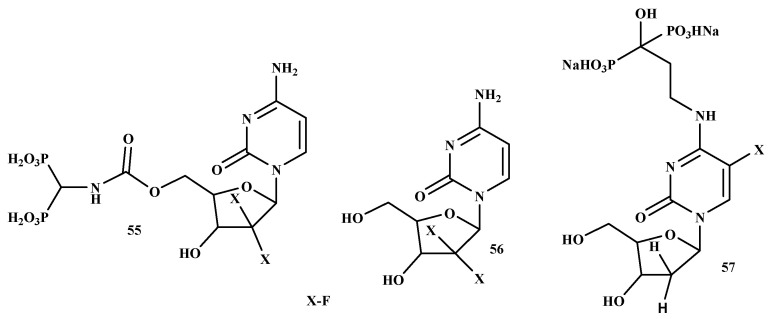
Structures of promising BP-conjugates with anti-lung cancer activity **55–57.**

An ideal chemotherapeutic drug designed for the treatment of bone metastases should specifically target the cancer cells in the bone, resulting in cytotoxicity to malignant cells while sparing normal cells, particularly in the bone marrow. Various efforts have been made to utilize BPs for the transportation/delivery of radiotherapy and/or chemotherapy based on their known affinity for bone [116]. El-Mabhouh et al. developed a drug delivery system using an improved BP (Figure 12) as a bone-seeking carrier to transport the chemotherapeutic drug, gemcitabine (Gemzar) (55) to bone lesions while preventing the normal tissues from the toxic effect of the drugs. Compound 55 is commonly utilized as an adjuvant or palliative drug for NSCLC, bladder, breast cancer, and pancreatic. Moreover, compound 55 exhibits a wide efficacy profile with other forms of cancer. Its broad therapeutic profile makes it an attractive antineoplastic agent for targeted chemotherapy applications [117]. Schott et al. prepared a new antimetabolite-BPs conjugate, 57. The IC_50_ concentration of the compound on Lewis lung carcinoma (LLC) exhibited an incubation time-dependent growth inhibition with higher sensitivity towards the tumor cells. The cytotoxic activity of compound 57 was confirmed by the cell viability test. To reach the IC_50_ for LLC cells, incubation of 72 h was required since the incubation of 24 h was not sufficient for the compound. However, the fundamental mechanisms of these promising novel antimetabolite-BPs conjugates remain to be evaluated in future experiments [118]. Table 6 depict a summary of the mode of action of bisphosphonate on different types of cancer.

## 5. Conclusion and Future Perspective

Bisphosphonates have been utilized as therapeutic agents for the treatment of osteoporosis, Paget’s disease, bone pain related to metastatic disease, hypercalcemia of malignancy, in diagnostic nuclear medicine, and targeted radiotherapy [134]. Therefore, the need for anti-osteoporotic agents that can be used for a prolonged duration with significant safety and efficacy is very important. To inhibit fractures in people with osteoporosis, BPs therapies that are effective in decreasing the risk of fractures and preventing osteoclast activity, inhibit osteoclast genesis and promote osteoclast apoptosis, are crucial. The results of vitamin D-bisphosphonate derivatives displayed stimulated bone matrix formation in dosages that did not increase calcium levels, indicating the existence of a therapeutic window for the treatment of bone diseases. The synthesized vitamin D-bisphosphonate compound prompted osteoid formation in vivo without elevating calcium levels. The bisphosphonate-conjugated estrogen displayed a preference profile in the bone due to its characteristic distribution pattern related to the natural estrogen. Therefore, bisphosphonate-conjugated estrogens have the potential to enhance patient compliance in estrogen treatment by decreasing the side effects and decreasing the frequency of drug administration.

BPs have been successfully used for the treatment of cancer-related bone disease and in reducing pain and skeletal complications. The skeleton is the most prevalent site to be affected by metastatic cancer. Prostate, lung, and breast cancers are responsible for the majority of skeletal metastases. This reflects both the high incidence and relatively long clinical course of these tumors [37,41]. BPs have also been utilized to decrease pain in multiple myeloma and reduce skeletal complications and the metastatic bone phase of a variety of solid tumors, for example, lung, prostate, and breast cancer. Some studies revealed the added beneficial clinical effect of BPs in cancer patients is associated with their anticancer activity. Some of the derivatives showed strong cytotoxicity on multiple myeloma cell lines and decreased the number of viable cells in a dosage-dependent manner. DEBP-Pt with high hydroxyapatite affinity showed bone resorption inhibitory effect and anticancer activity. The results suggest the potential of DEBP-Pt as a drug for metastatic bone cancer. Some BP-conjugates such as compound 47 could be delivered to the bone and demonstrated anticancer activity and prolonged survival, making it a promising therapy for TIBD. Compound 47 is one of the BP-conjugates in clinical trials. 5-Fluoro-2′-deoxyuridine-alendronate treatment in mice with bone metastases resulted in bone formation, inhibition of bone resorption, and inhibition of tumor growth. Long-term in vivo studies are warranted to determine the safety, effects on bone modeling and remodeling, and to further elucidate the cellular and molecular mechanisms leading to inhibition of tumor growth [88]. Linking BPs with conventional therapies for improved targeting and cytotoxicity in the case of cancer cells has the potential to provide synergistic effects for improving many treatments.

## Figures and Tables

**Table 1:** The structures of 3 generations of bisphosphonates.

1st Generation	2nd Generation	3rd Generation
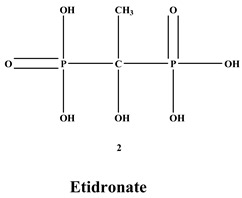	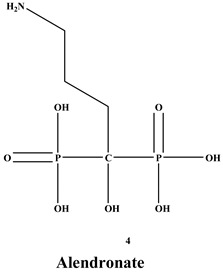	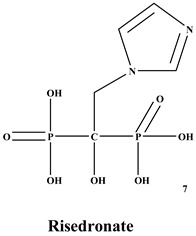
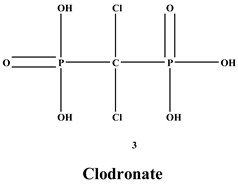	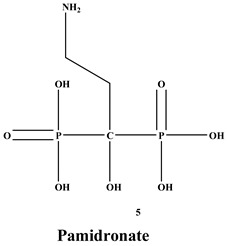	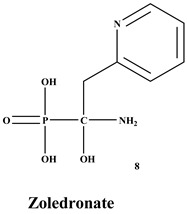
	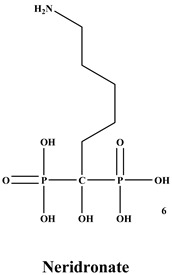	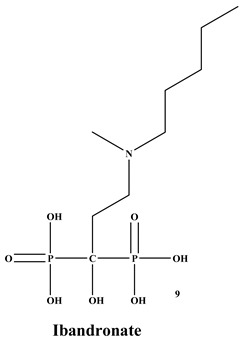

**Table 2:** Anti-proliferative and cytotoxic effects of the bone-targeted proteasome inhibitors.

IC_50_ (nM)					
Cell Line	PS-341	PS-341-BP-1 (24)	PS-341-BP-2 (25)	MG-262	MG-262 BP (26)
5TGM1	6.78 + 0.57	7.59 + 0.68	9.39 + 0.78	9.18 + 0.54	4.89 + 0.12
RPMI 8226	9.50 + 0.48	10.18 + 0.87	11.51 + 0.92	13.76 + 0.89	12.47 + 0.87

**Table 3:** Cytotoxicity of the DEBP-Pt, compound **29** against Various Human Tumor Cell Lines.

Cell Lines	IC_50_ (mol/L)
HLC-2	2.6 × 10^−5^
HCC1954	2.8 × 10^–5^
MCF-7	2.6 × 10^–5^
K562	4.7 × 10^–6^

**Table 4:** Effect on Bone Resorption Activity of Osteoclast.

Substance	IC_50_ (mol/L)
DEBP-Pt	2.8 × 10^–−6^
Cisplatin	1.1 × 10^–5^

**Table 5:** In vitro evaluation of breast cancer cell line.

	MCF-7		MDA-MB 231		OvCa-3		OvCa-29	
	IC50	IC90	IC50	IC90	IC50	IC90	IC50	IC90
5-fluorouracil	25.5	44.2	24.4	52.1	1.2	6.3	10.7	30.0
Zoledronate	8.9	42.9	6.4	39.1	1.6	6.0	3.0	7.2
5-Fluoro-2′-deoxyuridine-alendronate	51.3	83.8	12.8	62.5	24.5	64.7	8.2	62.5
Alendronate	55.9	100.7	32.3	69.7	12.7	39.8	16.6	38.2
5-Fluoro-2′-deoxyuridine-alendronate-aledronate	45.0	73.3	44.9	79.6	59.9	123.0	40.1	69.5

**Table 6:** Mode of action of BPs in breast, prostate, and lung cancer.

Types of Cancer	Mode of Action of BPs	References
Breast	- Inhibits proliferation of breast cancer cells, inhibits FPPS of the mevalonate pathway and inhibits GGPPS.	[119,120]
	Shows high affinity to bone matrix hydroxyapatite breast cancer.	[121]
	- Induces apoptosis by preventing ATP-dependent enzymes and prevents their absorption capacity.	[122]
	- Prevents breast cancer cell adhesion to the bone in vitro. - Inhibits the development and capability of cultured human breast cancer cells. - Induces loss of cell capability and DNA fragmentation in MCF-7 cells.	[123]
	- Prevents recurrence in postmenopausal women only.	[124]
	- In vitro, prevents tumor cell invasion, adhesion, migration, proliferation, and induces tumor cell apoptosis.	[125]
	- Improves the capability of antineoplastic agents to prevent breast cancer cell invasion.	[126]
	- Induces MCF-7 cell death and inhibit MCF-7 cell growth.	[127]
Prostate	- Has exhibited to apply a direct cytostatic and pro-apoptotic impact on PCa cell lines in vitro. - Inhibits cell invasion and adhesion through a decrease of matrix metalloproteinase appearance. - Prevents testosterone-prompted angiogenesis in a castrated animal model.	[128]
	- Prevents proliferation and induce apoptosis of prostate cancer cell lines in vitro. - In vitro studies of PC-3, LNCaP, and Du145 cell line, it prevents proliferation, induces apoptosis, decreases cell viability, and causes cell-cycle arrest.	[129]
	- Can down-regulate the expression of Bcl-2. - Induce apoptosis in prostate cancers. - Prevents proliferation markers, destroying the proliferation of tumors.	[130]
Lung	- Prevents cell proliferation in SCLC and NSCLC cell lines. - Uses its anti-proliferative impact against NSCLC by the initiation of cellular apoptosis via the small GTP-binding proteins related signal transduction pathway.	[115,131]
	- Prevents cancer cell cycle progression of NSCL carcinomas. - Induces cancer cell apoptosis in osteosarcoma, melanoma, and mesothelioma.	[125]
	- Improves cancer cell apoptosis, yields synergistic anticancer effects.	[132]
	- Prevents the action of osteoclasts and induces osteoclast apoptosis. - Exhibits prevention of the mevalonate pathway, regulation of immune response, and affects tumor signaling pathways and anti-angiogenesis.	[133]
